# Vulvar cancer during pregnancy and/or breastfeeding: a report of five cases from a single center study at the University Hospital of Düsseldorf

**DOI:** 10.1186/s12884-022-04448-x

**Published:** 2022-03-15

**Authors:** Andreas Suhartoyo Winarno, Tanja Natascha Fehm, Monika Hampl

**Affiliations:** Department of Obstetrics and Gynecology, The Heinrich Heine University Hospital of Duesseldorf, Moorenstraße 5, 40225 Duesseldorf, Nordrhein-Westfalen Germany

**Keywords:** Vulvar cancer, Pregnancy, Breastfeeding, Sentinel lymph node groin biopsy, Inguinofemoral lymphadenectomy

## Abstract

**Background:**

The incidence of vulvar cancer (VC) in pregnancy is unknown due to its rarity; between 1955 and 2014 only 36 case reports were reported worldwide. Underreporting may also be a contributing factor to the unknown incidence of VC in pregnancy. The aim of this study was to analyze the diagnosis, treatment and outcome of vulvar cancer cases diagnosed during pregnancy and/or breastfeeding.

**Case presentation:**

Patient 1 was diagnosed at 18 weeks’ gestation (WG) with Grade 2 VC (pT1a, pN0, 0/4 sentinel lymph nodes biopsy (SLNB) involved) and was treated by having the tumor resected (R0). She is currently recurrence-free at 4 years post-diagnosis.

Patient 2 was diagnosed at 7 WG with Grade 2 VC (pT1b, pN1a, 1/17 SLNB, R0) and was treated during the first trimester and during the second trimester with SLNB. She is currently recurrence-free at 5 years post-diagnosis.

Patient 3 was diagnosed at 30 WG with Grade 2 VC (pT1b, pN0, 0/5 SLNB, R0). She subsequently experienced a number of local recurrences postpartum that were managed by resection and is currently recurrence-free at 3 years post-diagnosis.

Patient 4 was diagnosed a VL later, at 14 months during breastfeeding, that was diagnosed as Grade 3 VC (pT1b, pN1a, 1/14 SLNB, R0). The patient is currently recurrence-free at 9 years post-diagnosis.

Patient 5 was not diagnosed during pregnancy, but was diagnosed with G3 VC (pT2, pN2c, 2/17 SLNB, R0) 8 months postpartum. The patient due to the extent of tumor involvement and lymph node metastasis, underwent chemoradiation therapy post-surgery. Despite adjuvant therapy, the patient progressed and developed bone metastases. Analysis of the tumour tissue revealed increased expression of PD-L1 (programmed cell death protein 1) indicating that the patient may have benefited from treatment with nivolumab to block the PD-L1 interaction; unfortunately the patient passed away at 24 months post-diagnosis before immunotherapy treatment could commence.

**Conclusion:**

Surgical resection and simultaneous SLNB in VC cases are considered safe during pregnancy, with comparable outcomes to non-pregnant women. Prompt diagnostic workup and treatment should never be delayed during pregnancy as delayed diagnosis could lead to tumour progression with fatal consequences.

## Background

In non-pregnant women, vulvar cancer (VC) is the fourth most common gynecological cancer, following cervical, uterine and ovarian cancer [1–6]. Common malignancies reported during pregnancy are breast cancer (46%) and hematological malignancies (18%). Other malignancies less commonly reported during pregnancy are malignant melanoma, brain tumor, thyroid, colon, ovarian, cervical and VC [5]. The mean age of patients with VC is 70 years, and the majority of tumors in this older population are due either to lichen sclerosis or old age. The occurrence of VC in younger women (less than 50 years of age) accounts for about 15% of all VC [6]. The incidence of VC during pregnancy is, however, unknown. Between 1955 and 2014, only 36 cases were reported in the literature reflecting an extremely rare likelihood of occurrence, although it is possible that underreporting or delayed diagnosis may contribute to the low incidence of VC reported during pregnancy [1]. In younger women, persistent infection of the squamous epithelium of the vulvar skin with high-risk human papillomavirus (HPV) types could result in the development of vulvar intraepithelial neoplasia (VIN) lesions with the subsequent risk of progression into malignant cells and invasive tumors if not promptly detected and treated. The progression rate of VIN into invasive cancer has been reported to be as low as 9%, and persistent high grade VIN can be removed surgically or treated locally [7, 8]. Hence close monitoring is advisable to enable early detection and diagnosis of VIN and also the prevention of progression of VIN to VC [1–6, 9].

There are two different pathways of vulvar cancer pathogenesis: lichen sclerosis or chronic skin diseases characterized by p53 mutated tumor cells (about 70% of all tumors) and HPV-induced vulvar cancer characterized by p16 overexpression [10]. HPV type 16, 31 and 33 are predominantly responsible for HPV-induced vulvar cancer, which occurs mainly in younger women with a mean age of 55 years [6, 11–13]. In contrast, lichen sclerosis-induced tumors more often affect older women, although exceptions are possible. Risk factors for HPV-induced pathogenesis are smoking and immunosuppressive conditions following organ transplantation or human immunodeficiency virus (HIV) infection. Verrucous carcinoma (originating from condyloma acuminata), basal cell carcinoma, melanoma, sarcoma, invasive Paget’s disease (adenocarcinoma) and Bartholin gland carcinoma are responsible for only a very small portion of all vulvar malignancies [14–17].

Physiological changes that occur during pregnancy, such as plasma volume expansion of up to 50-60% in singleton pregnancy, increased blood circulation, weakened maternal immune system, increased expression of cancer-related inflammatory cytokines and cancer metastasis to the fetal-placental tissue have been reported [1, 10]. These changes could potentially escalate the growth and spread of malignant cells [1]. Due to its rare occurrence, management of VC in pregnancy is not well informed and based mainly on available case reports [1–9]. The standard treatment of VC in pregnancy has not been well developed into current guidelines [4, 6, 11].

VC in pregnancy is rare, and there are currently no clear recommendations or guidelines on how VC diagnosed during pregnancy should be managed and treated.

A retrospective single center analysis was performed by the Oncology Unit of the Department of Obstetrics and Gynecology at the University Hospital Duesseldorf, which is specialized in the treatment of vulvar cancer cases, to highlight the importance of prompt management of VC and to guide treatment options and provide management recommendations for obstetricians/gynecologists and oncologists [18].

This is a retrospective, single center analysis of all women diagnosed with VC during pregnancy or breastfeeding at The University Hospital of Duesseldorf in Germany between 2004 and 2019. All vulvar cancer patients seen in the dysplasia clinic had been registered by diagnosis into the medical hospital documentation system. Pregnant or postpartum women diagnosed with VC were identified, and a retrospective review of their medical records was performed. Most of the identified cases were under close follow-up by the dysplasia unit according to the recommended schedule.

Five women, in the Obstetrics and Gynecology Department at The University Hospital of Duesseldorf, were identified as being diagnosed with VC during pregnancy and/or breastfeeding between 2004 and 2019. Informed consent for data analysis and publication of findings was obtained from all selected patients. Gestational age was defined as follows: first trimester being < 14 weeks’ gestation, second trimester being < 28 weeks’ gestation and third trimester being ≥28 weeks’ gestation. Full term pregnancy is defined as ≥37 weeks’ gestation.

Patients with a suspicious vulvar lesion (VL) first underwent a punch biopsy to provide tissue for an initial histological diagnosis. In confirmed cases of VC, the tumor was resected locally with >3 mm tumor-free margins or a partial or total vulvectomy in addition to sentinel node dissection was performed. Regional flaps for wound closure were indicated in accordance with German guidelines [6].

The Department of Nuclear Medicine at The University Hospital Duesseldorf had previously established a SLNB protocol for procedures undertaken during the 2nd and 3rd trimester of pregnancy. The protocol (described here as the ‘short protocol’) used a reduced dose of technetium (Tc-99^m^) nanocolloid to visualize SLNs, which was administered approximately 2 h’ prior to surgery. The dose of Tc-99^m^ was considered small and safe for use during pregnancy with a maximal dose of 50 MBq, which resulted in uterine exposure of about 1 mSv. If the Tc-99 m application was lower than 50 MBq, the uterine exposure would also be less than 1 mSv [19–22].

All patients underwent peritumoral intradermal injection of Tc-99^m^ positioned at three, six, nine and twelve o’clock using a 27-gauge needle. An hour after the injection, a planar lymphoscintigraphy was performed with anterior and lateral static views. An abdominal shield was used during lymphoscintigraphy in pregnant women to protect the fetus. During surgery, a handheld gamma probe (Neoprobe GDS, BT Devicor Mammotomo, Cincinnati, OH, USA) was used to identify labelled groin lymph nodes bilaterally. In the case of histologically proven SLN metastasis, complete inguinofemoral lymphadenectomy (IFL) was performed separately with additional patient consent. Pelvic node dissection was not performed during pregnancy, although it was eventually indicated in accordance with German guidelines following termination of pregnancy. The indication consisted of either having more than two metastatic nodes or one metastatic node > 5 mm or with extracapsular spread. In cases where there is a clinically high suspicion of groin metastasis on primary diagnosis, an IFL was performed without prior SLNB.

Prior to any surgical procedure, a prophylactic single course of betamethasone 2 × 12 mg for fetal lung maturation (LM) was administered to prevent respiratory distress syndrome in the preterm period between 24 and 34 weeks’ gestation following written consent.

Pathological examination was performed by staff of the Department of Histopathology at The University Hospital of Duesseldorf. A standard protocol for sentinel lymph node preparation was established, which involved using frozen sections of intraoperatively suspicious lymph nodes, staining with hematoxylin and eosin, and subsequent ultra-staging and immunohistochemistry using three sections per 5 mm.

VC is classified according to its histopathological features (p), tumor (T), nodal (N), sentinel lymph node (SLN), metastasis (M), lymph vessel (L), blood vessel (V), perineural invasion (P), grading (G), and resection status (R). For tumor staging, the FIGO-classification (federation Internationale de Gynecologie et d’Obstretrique (FIGO) for tumor staging was used as well as the Union of International Cancer Control (UICC) tumor-node-metastasis (TNM) classification, 6th edition [20, 21].

An interdisciplinary approach was used to determine an appropriate management and treatment plan for patients. The tumor board consisted of a gynecologic oncologist, radiologist, radiotherapist, histopathologist and hematologist oncologist.

## Case presentations (Table 1)

### Patient 1

A 32-year-old woman (163 cm, 57 kg), gravida (G) 1, presented at 18 weeks’ gestation with a suspicious vulvar lesion of the right labia minora. Tissue biopsy revealed invasive squamous cell carcinoma, with questionable depth of infiltration. She had a history of infection with high-risk HPV in 2016 and VIN 1 in 2014 and VIN 2 in 2015. She received secondary HPV vaccination in 2011. Complete removal of the lesion with SLNB (suspicion of more than microcarcinoma clinically) following administration of 20 MBq Tc-99^m^ was performed at 19 weeks’ gestation. The tumor diameter was measured at 10 mm with a depth of infiltration measured at 729 μm. Final histopathology results revealed a microcarcinoma tumour stage pT1a, pN0 (0/4 SLN), L0, V0, Pn0, G2, R0. The hospital tumor board recommended regular check-ups for follow-up. Due to persistent itching and burning sensation after surgery, a second biopsy on the left labia minora was performed, revealing VIN 2. Treatment with laser vaporization was not performed until 32 weeks’ gestation to ensure completion of fetal lung maturation. The baby was delivered via caesarean section (CS) due to pathologic fetal cardiotocography (CTG) detected at 42 weeks’ gestation. The patient was monitored with regular check-ups and assessment for VIN, with any recurrent VIN detected being managed and treated accordingly. Four years later, the patient delivered a second full-term, healthy baby via CS, and is currently recurrence-free 4 years post-VC diagnosis.

**Table 1:** Management and outcome of the 5 patients with vulvar cancer in pregnancy / breastfeeding period

	Punch Biopsy for diagnosis	Tumor stage	Local resection	Time of sentinel lymphadenectomy	Complete / radical groin dissection	Follow up (years)
**Patient 1**	18th weeks GA	1G, VC pT1a pN0 L0 V0 Pn0 G2 R0	19th weeks GA	19th weeks GA	Not performed	4
**Patient 2**	7th weeks GA	2G, 1P, pT1b, pN1a (1sn), V0, Pn0, G2, R0	8th weeks GA	19th weeks GA	20th weeks GA ipsilateral only	5
**Patient 3**	30th weeks GA	1G, pT1b, pN0 (0/5sn), L0, V0, Pn0, G2, R0	31st weeks GA	31st weeks GA	Not performed	3
**Patient 4**	14 months in breastfeeding period	2G, 2P, pT1b, pN1a (1/14) (sn), L0, V0, Pn1, G3, R0	14 months in breastfeeding period	14 months in breastfeeding period	Performed bilaterally	9
**Patient 5**	8 months in breastfeeding period	G2, P2, pT2, pN2c (2/17), L1, V1, Pn1, G3, R0 FIGO III c	8 months in breastfeeding period	Not performed	8 months in breastfeeding period incl. Pelvic LNE	died

### Patient 2

A 30-year-old woman (170 cm, 72 kg, gravida (G) 2, para (P) 1) presented at 7 weeks’ gestation with a 3-week history of persistent itching, pain and vulvar ulceration. Tissue biopsy revealed keratinized invasive squamous cell carcinoma pT1a induced by HPV 16. Complete tumour removal was performed at 8 weeks’ gestation with wound closure facilitated by use of a transpositional flap. Tumor diameter was measured at 21 mm with a depth of infiltration measured at 2 mm. Under the directive of the tumor board, she was scheduled for SLNB at 19 weeks’ gestation, during which she was administered 34 MBq Tc-99^m^ on the morning of surgery. Unfortunately, the final histopathological report showed a 5.5 mm metastatic SLN in her right groin. Therefore, bilateral inguinofemoral lymphadenectomy (IFL) was performed at 20 weeks’ gestation. There was no further groin node metastasis (0/16). The final histopathology results revealed a tumor stage pT1b, pN1a (1/17 SLN [0/9 right and 0/7 left]), L0, V0, Pn0, G2, R0. The patient’s baby was delivered via CS at term. She subsequently had an uneventful second pregnancy and delivered her full-term baby via CS. Upon her most recent examination at our clinic, 60 months after initial diagnosis, the patient showed no sign of recurrence. Five years later, she delivered her third full-term baby via CS.

### Patient 3

A 32-year-old woman, G1 (112kg, 168 cm) was referred by the local obstetrician with VL at 30 weeks’ gestation. She had a history of conization in 2009 due to high**-**risk HPV-related cervical dysplasia. Tissue biopsy revealed VC of the left labia minora**.** After administration of 15 MBq Tc-99^m^ on the morning of surgery, complete tumor resection and SLNB of both sides of the groin was performed. Tumor diameter was measured at 14 mm with a depth of infiltration measured at 2.6 mm. No lymph node metastases were found, resulting in the final tumor staging of pT1b, pN0 (0/5 SLN), L0, V0, Pn0, G2, R0. The patient delivered her full-term baby via CS. Six months later, she was diagnosed with persistent VIN 3, which was treated with laser excision. A year later, another local recurrence was identified and removed from the left introitus vagina that was histologically defined as microcarcinoma rpT1a, pNx, L0, V0, Pn0, G2, and R0. The advice from the tumor board was regular check-ups every 3 months. At her last follow-up in our clinic 3e years following initial diagnosis, she had maximal VIN 1 and is undergoing continued monitoring with regular check-ups.

### Patient 4

A 36-year-old woman, G2, P1 (164cm, 56 kg) was referred by the local obstetrician with VL at 23 weeks’ gestation. Tissue biopsy showed low-grade chronic inflammation, reactive squamous cell hyperplasia and hyperkeratosis. Fourteen months later, during breastfeeding, a recurrent VL was biopsied with the diagnosis of non-keratinized squamous cell carcinoma located at the right labia minora with extension close to the clitoris. After administration of 120 MBq Tc-99^m^ on the morning of surgery, local resection and SLNB were performed. Tumour diameter was measured at 12 mm with a depth of infiltration measured at 7 mm. The tumor was staged as pT1b, pN1a (1/2 SLN), L0, V0, Pn1, G3, R0. A 3 mm left-sided SLN metastasis with extracapsular tumor cells was identified, and bilateral IFL was performed. The remaining groin lymph nodes removed were negative (*n* = 12), resulting in final tumor staging of pT1b, pN1a (1/14 SLN), L0, V0, Pn1, G3, R0. The patient received radiotherapy on her left groin due to identification of the extracapsular tumor cells. She delivered a healthy third child spontaneously 7 years post-diagnosis. Patient has undergone regular follow-up for up to 9 years post-diagnosis with no sign of recurrence.

### Patient 5

A 39-year-old woman (167 cm, 67 kg), G2, P2 was referred by the local gynecologist 8 months after spontaneous delivery, with VL at the anterior fourchette with 1 cm infiltration of urinary meatus externus. According to the patient, the VL was present during pregnancy, however no further assessment was made. Tissue biopsy showed invasive keratinized squamous cell carcinoma, G3. She was infected with high risk HPV at the cervix, although HPV DNA in a vulvar swab was not detected. Due to the extent of the tumor and clinical suspicion of groin lymph node metastases, computed tomography (CT) scan and magnetic resonance imaging (MRI) were performed. These imaging assessments showed tumor infiltration of the clitoris and distal urethra, with potential right groin, pelvic ring involvement and right retroperitoneal lymph node metastasis, with no other further signs of distant metastasis. Therefore, complete removal of the vulvar tumor, part of the urethra, IFL of both sides of the groin and pelvic lymph node dissection were performed. Tumour diameter was measured at 52 mm, with vaginal and urethral infiltration including invasion of perineural tissue, lymph vessels and veins. The depth of tumor infiltration could not be determined. Bilateral groin lymph node metastases were reported; a 14 mm lymph node metastasis with extra-capsular tumor spread on the right side and a 14 mm lymph node metastasis without extracapsular spread on the left side. Extracted pelvic lymph nodes were metastasis free (0/6). Histology results showed no fat tissue or pelvic ring bone metastasis.

The final diagnosis for patient 5 was VC staged as pT2, pN2c (2/17 SLN), L1, V1, Pn1, G3, R0, FIGO IIIc. The tumor board suggested chemo radiotherapy to both sides of the groin with six cycles of cisplatin. Fourteen months later, she suffered from pathological fracture of right acetabulum, trochanter, and pubic bone, with involvement of adductor longus of obturators muscle internus, right iliac externa venous infiltration, edema in both legs and severe pain. These pathologies were due to growth of a malignant tumor in her pelvis (shown in CT scan). The tumor board recommendation was for further radiotherapy and chemotherapy with cisplatin and 5-fluorouracil (FU), with the addition of denosumab and hyperthermia (individual treatment decision).

Twenty-one months later, the patient suffered from local recurrence in her right peri-urethral region, progression of metastasis to ischial bone and venous thrombosis of right femoral and external iliac veins. Subsequent tumor tissue testing was positive for PD-L1 expression, and an immune checkpoint inhibitor (nivolumab) was recommended and approved by the patients’ health insurance as an individual treatment option. Unfortunately, she was unable to commence on immunotherapy due to the rapid progression of cancer and metastasis, and passed away 24 months following initial diagnosis.

## Discussion

Some obstetricians and gynecologists believe that certain invasive procedures should be avoided during pregnancy as the risk of profuse bleeding is increased during pregnancy [23]. Furthermore, medications used in general anesthesia are believed to be harmful to the fetus and/or the mother (pulmonary edema). On the contrary, many invasive operations have been performed in fetal surgery, for example for conditions such as spina bifida, with very low complication rates. A slight left tilt of the operation bed could reduce the risk of venous compression and relaxation medication can be used to help regulate blood circulation to the fetus. Close monitoring during intraoperative and postoperative procedures (continuous electrocardiography, systemic arterial and central venous blood pressures, oxygen saturation, urine production, bi-spectral index, extravascular lung water and body temperature measurements) are necessary to avoid complications [24]. Moreover, the placenta provides a good natural barrier for protecting the fetus from foreign substances; the significant reduction of drug bioavailability in the blood of the umbilical cord following antibiotic treatment has been reported due to placental barrier [25].

There are also many medications that have been proven to be safe to use in pregnancy, including chemotherapy agents such as Taxol and 5-Fluorouracil [2, 4, 5].

With any complaint of persistent itch, burning sensation, pain and/or ulceration, physicians must not hesitate to perform an immediate biopsy to rule out the possibility of a precancerous invasive lesion, including in pregnant women [1–9]. The SLNB procedure should not be offered to pregnant women under 14 weeks gestation and the procedure should be performed with lower doses of radioactive Tc-99^m^ nanocolloid, as per the ‘short protocol’ discussed previously, in order to minimize the risk to the fetus. The half-life of Tc-99^m^ is 6 h. The administration dose of less than 100 MBq Tc-99^m^ is considered safe for the fetus, with an estimation that fetal exposure is 1000 times lower than the administration dose [4]. In our center, Tc-99^m^ administration with a dose as low as 50 MBq was sufficient to detect sentinel lymph nodes, due to physiological changes of the lymphatic system during pregnancy. Physiological changes such as lymphangiogenesis due to vascular remodeling, mechanical compression in parallel with the increase in uterine size, flow reduction of lymphatic drainage, and retention of sodium and water may all play an important role in labelling the SLN within the groin [26, 27]. It seems that physiological changes during pregnancy permit Tc-99^m^ to work at a lower dose optimally. According to the gynecological group study from Levenback et al., the false negative value of SLNB was about 8.3% and the recurrence rate of metastasis was 2.7% [28]. In our study, we showed that the possibility of contralateral metastasis in non-SLN cases was about 22% (*n* = 4/18) after initial unilateral SLN metastasis [29]. Four of the five patients analyzed in our report had neither false negative results nor did they present with groin recurrences during their follow-up period. However, due to the small number of cases in our report, conclusions regarding the efficacy of low dose administration of Tc-99^m^ levels in labelling the SLN of the groin cannot be drawn, and further research is required to provide statistically meaningful data.

Stacker and colleagues conducted a lymphatic study in pregnant women that showed high expression of growth factors such as VEGF-C and VEGF–D in various human tumor cells were associated with lymphatic invasion, metastasis and in some cases with very poor prognosis of the patients [30]. At the time of presentation of patient 5 at our clinic (8 months postpartum) initial investigations showed a 5.2 cm ulceration suspicious of invasive cancer with the involvement of urethra and introitus. CT scan and MRI imaging revealed suspicion of lymph node metastasis in both sides of the groin, and involvement of retroperitoneal lymph nodes and the pelvic ring bone. Upon surgery, neither bone nor pelvic lymph node metastases could be confirmed. However, the rapid disease progression post-surgery despite adjuvant chemoradiotherapy with recurrence in pelvic bone and pelvic nodes suggested that the delayed diagnosis in this patient, without biopsy of the suspicious lesion due to pregnancy, could have led to extended lymph node metastasis and the fatal outcome for this young woman.

Methylene blue is an alternative method to Tc-99^m^ used to identify SLNs. However, due to its history of inducing anaphylactic shock, the use of methylene blue during pregnancy is prohibited. In standard protocols, lymphoscintigraphy should be performed following sentinel labeling of groin lymph nodes. The threshold for fetal damage during the imaging procedure is advised to be no greater than 100 mGy. Fetal radiation exposure to X-rays would be significantly reduced to less than 0.1 m Gy with the use of an abdominal shield. However, the lymphoscintigraphy does not necessarily to be performed. This means that after sentinel labeling of groin LNs has been done, the patient underwent directly to surgical LN removal. The detection of groin SLNs was using a handheld gamma probe. Prompt nodal removal can reduce the chance of systemic exposure, even though fetal exposure is considered low when Tc-99^m^ is injected locally in the peritumoral region [1–5, 9, 20, 21].

Delivery mode is another factor to consider after VC surgery and should be evaluated on a case-by-case basis, depending on probability of vulvar wound dehiscence and/or degree of scar tissue stenosis on the introitus [1, 4, 5].

If there is any indication to receive radiotherapy, it should be postponed - radiotherapy should be started following the birth of the baby.

A systematic review showed that the time interval from first medical visit to first diagnosis of VL was more than 8 weeks (62.5%). The first reason of delayed diagnosis is low suspicion of VC due to its rare occurrence in younger-aged women (70%), the second reason is noncompliance of patients (30%), and the third reason is the potential risks of a vulvar procedure resulting in feto-maternal complications during pregnancy. In comparison to all gynecological cancers in pregnancy, VC is in fact considered to have the least possible complications in patients undergoing biopsy and/or operation [1, 4, 5].

**Fig. 1:**
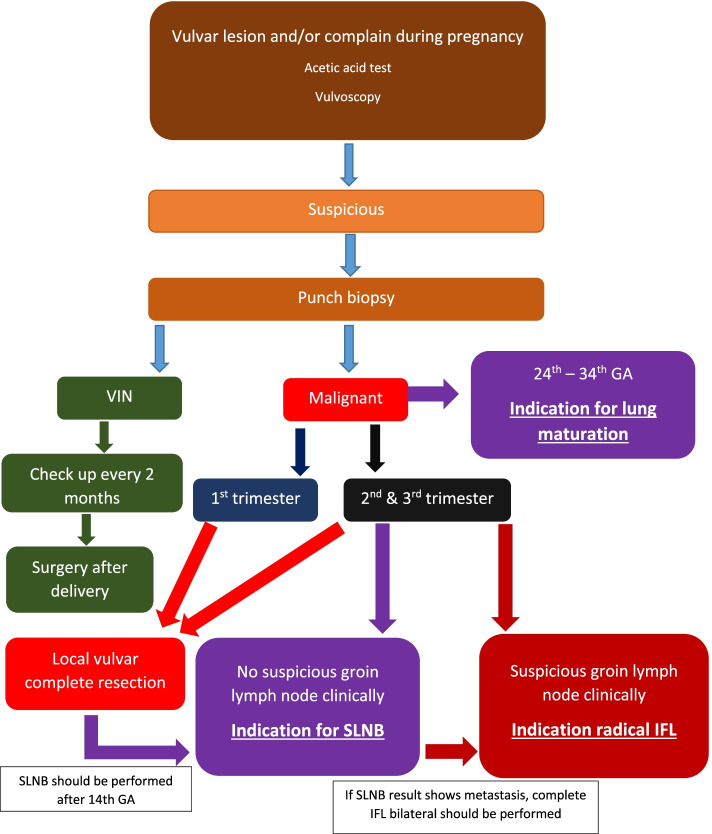
showed our recommendation for the management of vulvar lesion in pregnancy. HPV = human papilloma virus; VIN = vulvar intraepithelial neoplasia; GA = gestational age; SLNB = sentinel lymph node biopsy; IFL = inguinofemoral lymphadenectomy. *SLNB and/or radical IFL should be performed after 14th weeks of gestational age

This study elucidates the importance of prompt management of VC in pregnancy (see Fig. 1). In the case of patient 5, she was underdiagnosed during pregnancy, and was not diagnosed until 8 months after birth. Delayed diagnosis during her pregnancy resulted in fatal consequences for tumor progression, complications and treatment failure. The outcome for patient 5 contrasted with that from patients 1, 2 and 3 where all three women delivered full-term babies and are alive 6 years after initial diagnosis. Treatment for VC in these women was performed in accordance with the current German guidelines including local resection of tumor in toto, local flap reconstruction if indicated, and [6] SNLB with dose reduction of Tc-99^m^ [18, 19].

In pregnancy, vulvar cancer is the fourth most common malignancy after the most frequently diagnosed breast cancer, followed by cervical cancer and malignant ovarian tumor. The management of breast cancer in pregnancy is well established. The incidence of breast cancer is approximately 1 in 2000 to 10,000 pregnant women, with a median age of 33 years [31]. According to Maggen et al., breast cancer dominates with 41% of diagnoses during pregnancy and 70% of diagnoses during the postnatal period [32]. Surgical treatment can be performed beyond 12 weeks’ gestation, including SLNB. Chemotherapy is quite safe in the second or third trimester, using agents such as 5-fluorouracil, doxorubicin, epirubicin, cyclophosphamide, docetaxel, paclitaxel or methotrexate. Chemotherapy should be stopped 3 weeks prior to delivery to reduce the possibility of febrile neutropenia, hyperbilirubinemia, respiratory distress syndrome and apnea. The use of trastuzumab should be avoided during pregnancy to avoid renal and pulmonary complications of the newborn [33].

The incidence of cervical cancer is approximately 1 in 5000 to 20,000 pregnancies [5, 33, 34]. Cervical cancer accounted for about 10% of cancer diagnoses during pregnancy [32]. Every suspicious lesion at the cervix should be investigated by a dysplasia unit via Pap smear/HPV test, colposcopy and biopsy. However, endocervical dilatation and curettage should be avoided. Conization should not be performed during pregnancy, except in the case of high grade CIN where there is suspicion of early invasive cancer, and it may be performed between 14 and 20 weeks’ gestation. Large conization of cervical malignancies up to Stage IA2 and IB1 including cerclage and lymph node dissection may be performed during pregnancy. Staging of pelvic lymph nodes can be performed until 22 weeks’ gestation [31]. After 22 weeks’ gestation, cervical cancer should not be treated surgically until after delivery. Chemotherapy in pregnancy for cervical cancer is considered quite safe, using cisplatin monotherapy, cisplatin with paclitaxel or alternative regimens such as cisplatin with vincristine [5, 34]. However, the study from Hecking et al. suggested that conization should not be performed during pregnancy due to increased risk of bleeding/miscarriage [33]. Previous discussions on the best mode of delivery for pregnant women diagnosed with cervical cancer has been controversial. However, there have been at least two reported cases suggesting vaginal transmission of cervical cancer cells to infants and impaired prognosis of the mother after spontaneous delivery. Indeed, nowadays caesarean section is the recommended mode of delivery [35].

The incidence of malignant ovarian tumor is approximately 1 in 12,000 to 100,000 pregnancies. A suspicious ovarian mass may be removed at the end of the first trimester or during the second trimester of pregnancy. The primary reason is to avoid torsion and secondly, histological examination is necessary for guide further treatment decisions. Radical surgery (tumor debulking) is to be done after delivery [5, 34]. Chemotherapy, using carboplatin and/or paclitaxel, is possible if administered during the second and third trimester (16-36 weeks’ gestation). Carboplatin is preferred to cisplatin due to the higher intrauterine growth restriction rate, preterm birth risks, oligohydramnion, respiratory distress syndrome and neonatal anemia associated with the use of cisplatin [5, 34].

## Conclusion

A vulvar biopsy performed in pregnant women is considered safe for the management of suspicious VL, along with surgical resection methods and facultative SLNB in cases of proven vulvar malignancy with comparable outcomes to that observed in non-pregnant women. Delayed diagnosis could result in fatal outcomes such as tumor progression, complications, and treatment failure, as observed in our study patient 5. Therefore, prompt diagnostic workup and treatment should never be delayed during pregnancy.

## Data Availability

Retrospective single center study at the department of O&G, UHD. The data cannot be shared publicly in according to data protection act (Datenschutzgesetz) of German regulation. The datasets used and/or analysed during the current study are available at UHD.

## Abbreviations

B: Biopsy
CS: Caesarean Section
CTG: Cardiotocography
CT scan: Computed Tomography scan
FIGO: International Federation of Gynecology and Obstetrics
FU: Fluorouracil
GA: Gestational age
HPV: Human Papilloma Virus
IFL: Inguinofemoral Lymphadenectomy (complete radical groin dissection)
MRI: Magnetic Resonance Imaging
rCS: Repeat caesarean section
SLN: Sentinel Lymph Node
Tc 99^m^: Technetium 99^m^
T, N, M, L, V, Pn, G, R: Tumor, Node, Metastasis, Lymph vessel, Blood vessel, Perineural invasion, Grading, Resection status
UICC: Union of International Cancer Control
UHD: University Hospital of Duesseldorf
VC: Vulvar Cancer
VIN: Vulvar Intraepithelial Neoplasia
VL: Vulvar Lesion
